# Evaluation of Cyantraniliprole and Other Commercial Fly Baits under Laboratory and Field Conditions

**DOI:** 10.3390/insects6040977

**Published:** 2015-11-23

**Authors:** Casey Parker, Rebecca Baldwin, Roberto Pereira, Philip Koehler

**Affiliations:** Department of Entomology and Nematology, University of Florida, 970 Natural Area Dr., Gainesville, FL 32611, USA; E-Mails: baldwinr@ufl.edu (R.B.); rpereira@ufl.edu (R.P.); pgk@ufl.edu (P.K.)

**Keywords:** house fly, *Musca domestica*, cyantraniliprole, fly control, fly bait

## Abstract

Laboratory and field trials were performed to evaluate the attractiveness and efficacy of commercial baits (cyantraniliprole; methomyl + (*Z*)-9-tricosene; dinotefuran + (*Z*)-9-tricosene; imidacloprid granular + (*Z*)-9-tricosene; and imidacloprid liquid + (*Z*)-9-tricosene). In choice tests; flies were most attracted to cyantraniliprole bait > dinotefuran + (*Z*)-9 > methomyl + (*Z*)-9 bait > imidacloprid granular + (*Z*)-9 bait > imidacloprid liquid + (*Z*)-9 bait. Significant degradation in bait efficacy was observed after two weeks of aging excluding imidacloprid granular; which began to degrade in field conditions after one week. Cyantraniliprole; the new fly bait active ingredient in Zyrox^®^; had the longest time to knockdown in the laboratory tests; but on susceptible flies; achieved 95%–100% knockdown within an hour of exposure. Zyrox^®^ was resistant to weathering for a week; and was more attractive to flies in the field when compared to methomyl + (*Z*)-9 bait.

## 1. Introduction

House flies, *Musca domestica*, are considered to be both agricultural and public health pests [1]. House flies develop in moist areas that contain organic matter such as manure, animal feed and others. This organic matter can accumulate quickly allowing for rapid house fly population growth. Livestock facilities are, therefore, an ideal breeding and development site for flies and high populations at these facilities can cause aversive livestock behavior [2]. A variety of different methods are used in attempts to control this persistent pest. Toxic fly baits have been used for decades and are one of the most widely used methods for fly control. Animal operations depend heavily on these baits [3], but the heavy reliance on them has led to house fly resistance in many areas [4].

Due to the annoyance to humans and animals, and consequent public health concerns, speed of kill is an important aspect of fly baits. Carbamates and neonicotinoids are the two most common classes of chemistry available in fly baits and have relatively quick modes of action. In contrast, cyantraniliprole has a slower mode of action, but represents a new class of chemistry for fly control [5].

Most commercially available fly baits like Golden Malrin^®^ (methomyl), Maxforce Granular^®^ (imidacloprid), Maxforce Fly Spot Bait^®^ (imidacloprid), and Quikstrike^®^ (dinotefuran), incorporate the sex attractant, (*Z*)-9-tricosene, which has been used in fly baits since it was isolated from the cuticle and feces of female house flies in 1971 [6]. Since then, numerous studies have shown that the incorporation of (*Z*)-9-tricosene increases fly catch [7,8,9] and it has become a standard component in toxic fly baits. In contrast with other commercially available fly baits, Zyrox^®^ (cyantraniliprole) incorporates a food matrix that acts as the bait’s attractant. Recent studies have shown that Zyrox^®^ is highly attractive to house flies in field studies even though it does not incorporate (*Z*)-9-tricosene [3].

The objectives of this study were to evaluate the efficacy of a bait that lacks the commonly used attractant, (*Z*)-9-tricosene, and also has a slower mode of action than other commercially available fly baits, and to evaluate the longevity of these different baits. In this study, time to knockdown and bait preference of flies is determined in laboratory studies and the attractance and efficacy of some baits is evaluated in a field scenario.

## 2. Experimental Section

### 2.1. Insect Rearing and Test Conditions

Insecticide-susceptible house flies, *Musca domestica* (Linnaeus), USDA Center for Medical, Agricultural and Veterinary Entomology Orlando Normal strain were used. The colonies were maintained using rearing procedures established by Hogsette [10]. Pupae were transported to the University of Florida Urban Entomology Lab (Gainesville, FL, USA) and held within screened cages until emergence. Adult flies were maintained at 26 ± 1 °C, 55% RH, a photoperiod of 12:12 (L:D) h, and given access to water and dry diet of powdered milk, egg yolk, and granulated sugar *ad libitum* [11]. For all laboratory assays, adult house flies (3–5 d old) were aspirated with the crevice tool of a handheld vacuum. When used in no-choice tests, flies were chilled in a 5 °C environment until immobile, then placed on a chilled aluminum tray for counting. For choice assays, adult flies were aspirated from the emergence cage and placed directly into the test cage. In the field trials, wild fly populations were observed from thoroughbred horse farms in Alachua and Marion Counties, FL, USA.

### 2.2. Fly Baits

Baits tested were commercially available and included: cyantraniliprole (Zyrox^®^, Syngenta Corporation, Wilmington, DE, USA), methomyl + (*Z*)-9 (Golden Malrin^®^, Wellmark International, Schaumburg, IL, USA), dinotefuran + (*Z*)-9 (Quikstrike^®^, Wellmark International, Shaumburg, IL, USA), imidacloprid + (*Z*)-9 (MaxForce Fly Spot Bait^®^, Bayer Environmental Science, Research Triangle Park, NC, USA), and imidacloprid + (*Z*)-9 (MaxForce Granular Bait^®^, Bayer Environmental Science, Research Triangle Park, NC, USA).

### 2.3. No-Choice Bioassay with Fresh and Aged Baits

Unstarved, mixed-sex, 3 to 5 d old house flies were counted into groups of 15. Flies were aspirated from colony cages and chilled on a cold tray for sorting before placement into 1 L Mason jars with cloth covers. Water was provided *ad libitum*. After a 1 h acclimation period, a polystyrene petri dish (35 mm × 10 mm) (Thermo Fisher Scientific, Waltham, MA, USA) containing 1 g of granular bait was placed in the Mason jar. All baits were used in the experiment plus a control with no bait. Imidacloprid liquid bait was mixed by adding 2.5 g of bait with 7.5 mL of water. Liquid formulation was sprayed onto filter paper and allowed to dry for 1 h before placement into the Mason jar. The experiment was repeated with aged baits. Baits were aged outdoors with partial sun exposure for one week in a petri dish. Flies were allowed to feed for two hours and knockdown was recorded each minute during the feeding time. Criterion for knockdown was the inability of the fly to right itself. Flies were discarded at the end of the 2 h study period. Knockdown data was corrected using Abbot’s equation [12], and data was arcsine square root-transformed before analysis. Six replicates were performed within the same day. Time to knockdown was analyzed using an ANOVA with treatment as the independent variable. Percent knockdown was analyzed with mean separation using a *T*-test with α = 0.05 [13].

### 2.4. Choice Bioassay

Approximately 150 unstarved, mixed-sex, 3 to 5-d old house flies were aspirated and directly released into a cage (0.74 m × 0.74 m × 0.91 m). Water was provided *ad libitum* and replicates were performed under laboratory conditions at approximately 23 °C. After a 1h acclimation period, covered, polystyrene petri dishes (100 mm × 15 mm) (Thermo Fisher Scientific, Waltham, MA, USA) containing 9 g of bait were added in an equidistant ring around the center water dish approximately 7 inches apart. A video camera was placed above the cage to record fly preference for each bait choice. Baits were uncovered, and flies were allowed to feed for 35 min. Analyses of the feeding preferences of flies were made by pausing recordings at 0.5, 1, 2, 5, 10, 15, and 30 min and recording the number of flies touching the bait or dish. At the 30 min reading for all replicates, greater than 75% of flies were being either physically affected by baits or had died. Four replicates were performed on separate days within a 36-day period. Fly feeding preference was analyzed using a one-way ANOVA [13] with bait as the independent variable.

### 2.5. Field Attractiveness Trial

**Figure 1:**
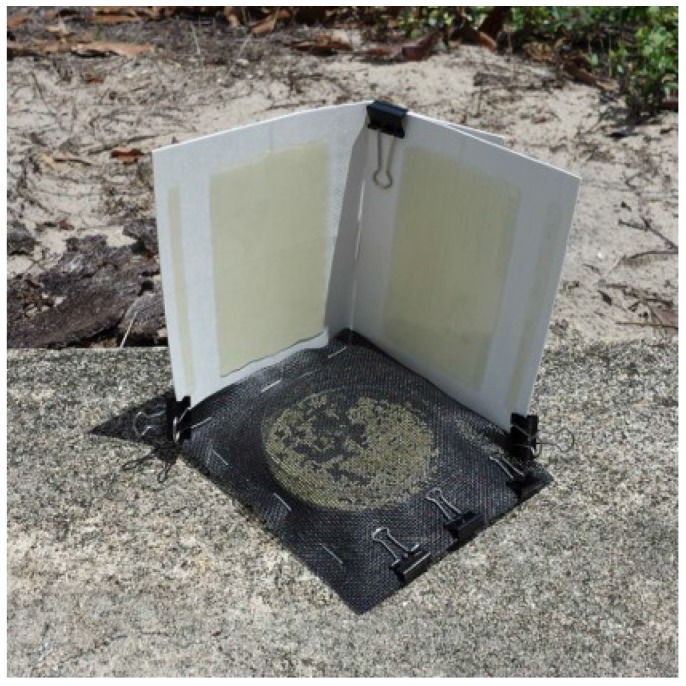
Field baited attraction evaluation device.

Cyantraniliprole and methomyl baits were selected to test field attractiveness. Methomyl bait has been used in fly control for decades and was therefore chosen as the standard to compare with cyantraniliprole bait. Field attractiveness trials were conducted at commercial equine barns in Ocala and Gainesville, FL, USA. Baited attraction evaluation devices (BAED) (Figure 1) were placed in areas of high fly activity such as shed rows, muck sorting areas, muck truck storage areas, and breezeways. BAEDs consisted of a screen covered (16 mesh fiberglass screen) 9 cm plastic petri dish secured between two 10.2 cm × 12.7 cm white cockroach sticky monitors (Woodstream Corp., Lititz, PA, USA) secured at 90° angles with the sticky side facing the treatment dish. Treatments included 5 g of cyantraniliprole or methomyl baits in the petri dish and an empty dish was used as a control. The experiment was replicated nine times using randomized complete block design. A replicate represents placement of the three different treatments at a location at a period of time. Locations were reused either at different times on the same day or on different dates within a period of three days. BAEDs were separated by 2–3 m. Treatments at the Ocala facility were left in place for 24 h and treatments at the Gainesville facility were left in place for 2 h to determine the proportion of files attracted to each bait. Flies on each BAED were counted and treatment was analyzed by a one-way ANOVA with treatment as the independent variable. Treatments were compared using the nonparametric Dunn method for joint ranking [13].

### 2.6. Field Efficacy

To determine the efficacy of selected baits in the field, testing was performed in three 36-stall thoroughbred horse barns in Marion County, FL. Testing was performed in areas with high fly activity: (1) feed rooms; (2) breezeways; (3) wash racks; (4) stalls; and (5) muck truck storage areas. Five replicates were made in a randomized complete block design with a single bait treatment and attractant located at each of the five treatment areas. Ocala Breeder’s Sales Performance Blend sweet feed (Ocala Breeder’s Farm Supply, Ocala, FL, USA) was used as the attractant. Baseline mortality fly counts were taken before the treatments were added. Immediately after the pre-treatment counts were taken, the baits and attractants were distributed uniformly across the treatment area. Each block contained one of the following three treatments: (1) control (68g attractant only); (2) cyantraniliprole bait plus attractant; or (3) methomyl bait plus attractant. Application sites were approximately 13 m^2^ and baits were applied according to label instructions at a rate of 113 g per 46.5 m^2^ (approx. 34 g of bait at each location). The bait was distributed after the baseline count and 60 min was allowed for flies to settle before post-treatment data collection. Counts of dead flies within the sample area were taken at 60 and 120 min post-treatment. Data was square root-transformed (x + 0.5) and analyzed using one-way analysis of variance (ANOVA) with treatment as independent variable followed by *T*-test for means separation [13].

## 3. Results and Discussion

### 3.1. No-Choice Bioassay with Fresh and Aged Baits

**Figure 2:**
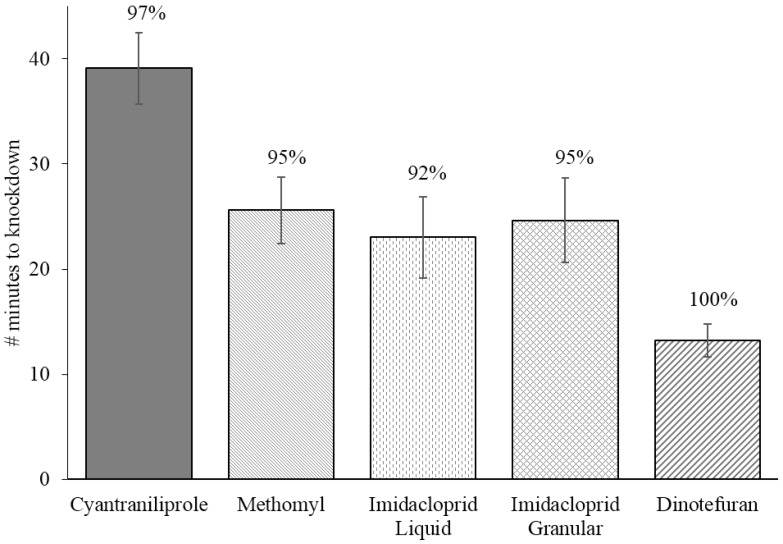
Mean (±SEM) time to knockdown of flies after ingestion of different fly baits. Four replications of 15 flies were allowed to feed on the baits in a no-choice bioassay for a total of *n* = 60 for each fly bait. Note: Percent knockdown of flies (numbers above bars) used in time to knockdown experiment are shown in Figure 3 as week 0.

The average time to knockdown for flies in minutes was 39.1 ± 3.4 (mean ± SEM) for cyantraniliprole bait, 25.6 ± 3.2 for methomyl bait, 23.0 ± 3.9 for imidacloprid liquid bait, 24.7 ± 4.0 for imidacloprid granular bait, and 13.2 ± 1.5 for dinotefuran bait (Figure 2). Flies exposed to cyantraniliprole bait had a significantly slower time to knockdown compared to flies exposed to all other baits. Imidacloprid liquid bait had a significantly slower time to knockdown than dinotefuran bait, but was not significantly different from imidacloprid granular and methomyl baits (*F* = 12.4; *df* = 4,276; *p* ≤ 0.0001).

**Figure 3:**
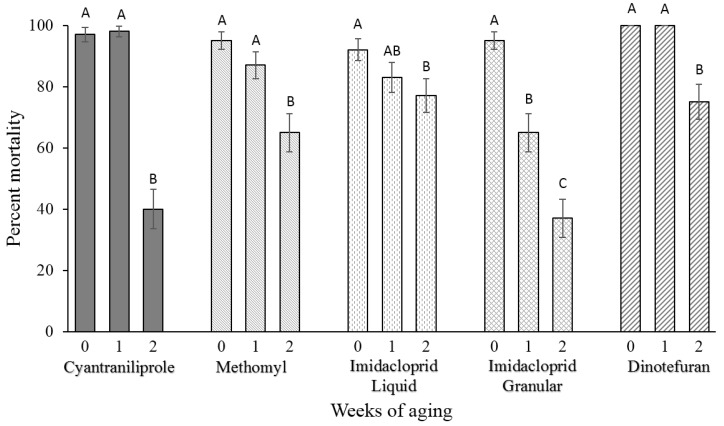
Percentage (±SEM) of flies killed by the fly baits over a two-week aging period. SEM bars with different letters for the same treatment represent a significant difference (*T*-test α = 0.05). Four replications of 15 flies were allowed to feed on the baits in a no-choice bioassay for a total of *n* = 60 for each fly bait during each week. Note: In control treatment, one fly died in one of the four replicates (1.7% control mortality).

Over the 2-week aging period, degradation of the baits was observed leading to a significant decrease in the percent knockdown of flies killed by different baits (*F* = 21.4, *df* = 2,885, *p* ≤ 0.0001) (Figure 3). Significant degradation of baits was not observed between week 0 and week 1, but was observed from week 1 to week 2 for cyantraniliprole (*F* = 62.5; *df* = 2177, *p* ≤ 0.0001), methomyl (*F* = 10.9; *df* = 2177; *p* ≤ 0.0001), and dinotefuran baits (*F* = 19.7; *df* = 2177; *p* ≤ 0.0001). Imidacloprid liquid bait did not show a significant degradation from week 0 to week 1 or from week 1 to week 2, but there was a significant difference from week 0 to week 2 (*F* = 2.5; *df* = 2177; *p* ≤ 0.0001). Strongest quality degradation was observed with imidacloprid granular bait, with significant decrease in the efficacy between weeks 0 and 1, and between weeks 1 and 2 (*F* = 25.0; *df* = 2177; *p* ≤ 0.0001).

Cyantraniliprole was slower in killing the flies due to its mode of action. Cyantraniliprole is a diamide, which acts on the ryanodine receptor in the muscle cells of flies. Consequently, the muscle cells continuously release calcium causing a slow paralysis [14]. This is a slow mode of action compared to carbamates and neonicotinoids. Carbamates are acetyl cholinesterase inhibitors [15] and neonicotinoids are nicotinic agonists that interact with the nicotinic acetylcholine receptor [16]. These different modes of actions account for the significant difference in the time to knockdown between cyantraniliprole and the other baits. It is important to note that flies did not recover from effects of active ingredients during the 2 h study period, but were discarded after the 2 h experimental time and could have recovered after being discarded.

A previous study presented results that contrast those presented in this article [3]. In that no-choice study, the UCR lab strain of flies took anywhere from 1 to 3 days to achieve 95%–100% mortality. In our no-choice lab study, 95%–100% knockdown was observed on fresh baits within an hour. This contrast in data could be due to a number of factors including fly strain and different bioassays. The Murillo study released 50 flies into 1 L test arena while in the presented study, 15 flies were released into a 1 L mason jar. This may have elicited feeding in a shorter period of time due to lower competition for food. Flies in the present study were also allowed to acclimate in the test jar for an hour before bait was placed while the Murillo study placed flies directly in the arena with baits.

Significant degradation of baits was observed even when the baits were allowed to age in a covered outdoor environment. Baits were exposed to natural temperature and humidity levels and absorbed water readily. The hydrophilic nature of the baits may have contributed to either a degradation of the active ingredient, the attractive agent, or both. Quick degradation of baits, even in protected conditions, demonstrates the need for repeated reapplication of baits for maximum efficacy.

### 3.2. Choice Bioassay

**Figure 4:**
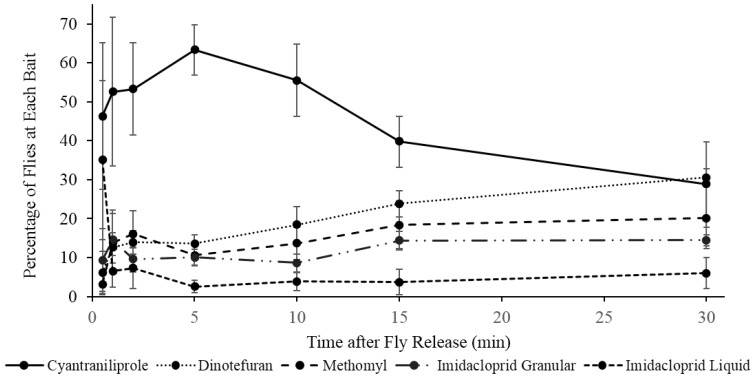
Percentage (±SEM) of responding flies on each fly bait at different time intervals over a 30-min period. Four replications of approximately 150 flies were performed (*n* = 4).

Significant differences were observed in the number of flies attracted to different baits. At the 0.5 (*F* = 1.2; *df* = 4, 12; *p* = 0.37) and 1-min (*F* = 1.4; *df* = 4, 12; *p* = 0.29) readings after initiation of bait exposure, no significant difference was observed in the attractance of baits, but starting after the 2-min reading, cyantraniliprole bait attracted significantly more flies than all other baits (*F* = 3.9; *df* = 4, 12; *p* = 0.03). The greatest differentiation in bait attractance occurred at 5-min when approximately 63% of the responding flies were on the cyantraniliprole bait and only 2.5% of flies were on imidacloprid liquid bait, which attracted significantly fewer flies than all other products (*F* = 24.0; *df* = 4, 12; *p* ≤ 0.0001). At the 15-min reading, cyantraniliprole bait continued to be more attractive than imidacloprid granular, methomyl, and imidacloprid liquid baits (*F* = 5.0; *df* = 4, 12; *p* = 0.013), but after the 30-min reading, there was no significant difference in the number of flies attracted to the different baits (*F* = 1.8; *df* = 4, 12; *p* = 0.19). On average, cyantraniliprole was the most preferred bait throughout the replications (*F* = 18.1; *df* = 4,127; *p* ≤ 0.0001) (Figure 4).

When given the choice of a variety of commercially available baits, the flies preferred cyantraniliprole. Bait preference has been evaluated in field scenarios, but this experiment shows clearly how the baits compare in terms of attractance over a short time. In situations where the user prefers a more attractive bait over a bait with a quick time to mortality, cyantraniliprole seems an ideal choice. Flies were observed spending a longer period of time on cyantraniliprole than the other baits. This may be due to the combination of a slow mode of action and the presence of food attractant in this bait. The other baits used in this study have a relatively quick mode of action and the feeding time was shorter.

Methomyl, imidacloprid liquid, imidacloprid granular, and dinotefuran baits all incorporate the sex pheromone, (*Z*)-9 tricosene into their respective bait formulations. The cyantraniliprole bait, on the other hand, incorporates a food matrix as the attractant and no sex pheromone. The difference in attractants may be one of the reasons for the cyantraniliprole bait’s success in controlling the flies and demonstrates that a fly bait does not need to contain the sex pheromone, (*Z*)-9-tricosene to be attractive to house flies. A previous study shows the addition of (*Z*)-9 tricosene to water did not significantly increase the number of flies attracted [17].

After 30-min, greater than 75% of the flies were dead or immobile, and most flies had fed on at least one of the baits and were being physically affected by the insecticides. An active ingredient with minimum immediate effect on the flies may be important to guarantee the consumption of sufficient bait to cause higher levels of mortality. Therefore, slow-acting baits can still be effective if they are preferred while flies are feeding.

### 3.3. Field Attractiveness Trial

**Figure 5:**
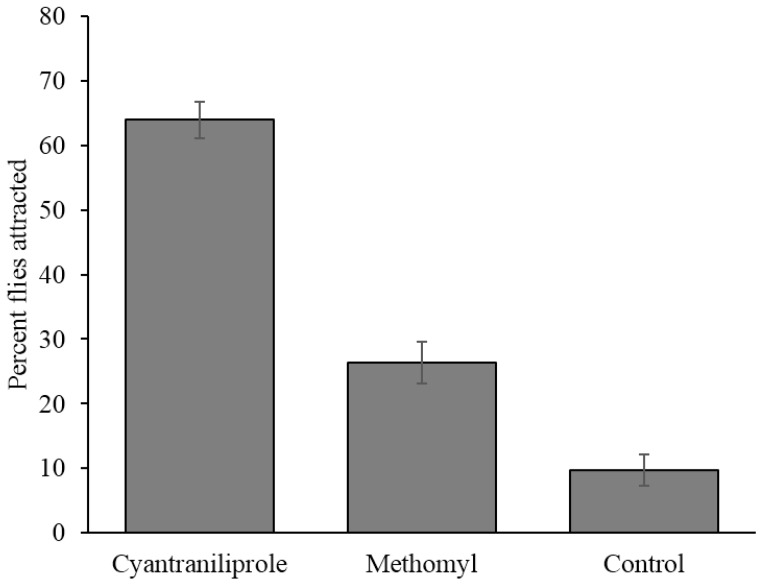
Percent (±SEM) of flies attracted to the different treatments in field study. Nine replications were done (*n* = 9).

Flies did not distribute themselves randomly among the treatments in the field and had a significantly higher proportion of them preferring cyantraniliprole bait (64.0%) over methomyl bait (26.3%) and the control (9.7%) (*F* = 95.1; *df* = 2, 24; *p* ≤ 0.0001) (Figure 5). The number of flies attracted to methomyl bait was not significantly different from the control (*p* = 0.10).

Previous results [3] show similar results with cyantraniliprole and spinosad (not evaluated in this study) being the most attractive baits in field studies. These authors also presented evidence that suggested resistance to methomyl, the active ingredient in Golden Malrin^®^, by flies used in the study. The populations between the two study areas are different, but methomyl has been used commercially for decades. Consequently, behavioral or physiological resistance may have been a factor that contributed to cyantraniliprole bait being more attractive than methomyl bait in the field. Behavioral resistance to a bait would decrease the attractiveness of that bait to flies. This could happen through an increased sensitivity to certain aspects of the bait which would cause the flies to avoid consuming the bait [18].

### 3.4. Field Efficacy

**Figure 6:**
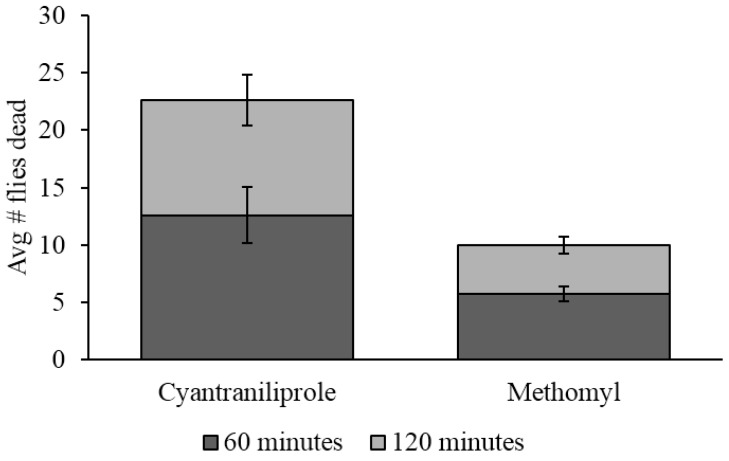
Average number of dead flies (±SEM) found in the sampling areas for each treatment after 60-min and 120-min. Three replications were done for a total of *n* = 3.

There was a significant difference between all baits when evaluated in the field. Cyantraniliprole bait caused a significantly higher mortality of flies than methomyl bait, which caused a significantly higher fly mortality than the control (*F* = 37.8; *df* = 2, 10; *p* ≤ 0.0001). On average, after 60 min, 12.6 dead flies were found in the sampling area for cyantraniliprole compared to only 5.8 for methomyl. After 120 min, 22.6 flies were found in the sampling area for cyantraniliprole compared with 10 for methomyl (Figure 6). Mortality was not observed in the control sampling areas.

During the field-testing of the baits, many dead flies were observed outside the sampling area for both cyantraniliprole and methomyl. However, numbers of cadavers outside the sampling area were higher for the cyantraniliprole than the methomyl treatment. Due to the slower mode of action for cyantraniliprole, flies exposed to this pesticide were likely able to disperse greater distances than flies affected by the methomyl bait. This behavior may have affected the number of flies observed in the sampling area. This is in contrast with flies affected by methomyl, which die quickly and, therefore, could not fly away from the established sampling area. Flies that have developed methomyl resistance would have been able to disperse from the test arena, therefore the low number of dead flies observed in methomyl testing arenas could be an indication of resistance in this field population. Despite the possibility of resistance and the limited number of active ingredients tested, the cyantraniliprole-containing bait was more effective than the methomyl-containing bait in this field trial.

## 4. Conclusions

Overall, the cyantraniliprole-containing bait, Zyrox^®^, outperformed a variety of other commercially available fly baits. It was the most attractive bait in preference studies both in the lab and field and was successful in killing more flies in the field when compared to methomyl bait only. Once ingested, cyantraniliprole has a slower time to knockdown than the other baits evaluated. However, cyantraniliprole only takes approximately 15–27 min longer than methomyl, imidacloprid liquid, imidacloprid granular, and dinotefuran baits. This is in contrast to previous studies that show cyantraniliprole bait taking 2–3 days to achieve similar levels of mortality [3].

In addition to the high attractance of flies to the cyantraniliprole-containing bait, the active ingredient is a reduced risk insecticide with little toxicity to vertebrates and other non-target organisms [15] making it a safer alternative to the other commercially available baits. In the past, carbamates and neonicotinoids have been the primary insecticides used for fly control. Resistance to different active ingredients in these chemical classes has been detected [4], which demonstrates the need for rotation to a new class of chemistry for fly control. The incorporation of cyantraniliprole bait into fly control programs would be beneficial in controlling fly populations and in resistance management.
